# Estimating the Effect of Healthcare-Associated Infections on Excess Length of Hospital Stay Using Inverse Probability–Weighted Survival Curves

**DOI:** 10.1093/cid/ciaa136

**Published:** 2020-02-12

**Authors:** Koen B Pouwels, Stijn Vansteelandt, Rahul Batra, Jonathan Edgeworth, Sarah Wordsworth, Julie V Robotham, Improving the uptake and SusTainability of Effective interventions to promote Prudent antibiotic Use and Primary care (STEP-UP) Team, Philip E Anyanwu, Aleksandra Borek, Nicole Bright, James Buchanan, Christopher Butler, Anne Campbell, Ceire Costelloe, Benedict Hayhoe, Alison Holmes, Susan Hopkins, Azeem Majeed, Monsey McLeod, Michael Moore, Liz Morrell, Koen B Pouwels, Julie V Robotham, Laurence S J Roope, Sarah Tonkin-Crine, Ann Sarah Walker, Sarah Wordsworth, Anna Zalevski

**Affiliations:** 1 Health Economics Research Centre, Nuffield Department of Population Health, University of Oxford, Oxford, United Kingdom; 2 National Institute for Health Research Health Protection Research Unit in Healthcare Associated Infections and Antimicrobial Resistance, University of Oxford, Oxford, United Kingdom; 3 Department of Applied Mathematics, Computer Science and Statistics, Ghent University, Ghent, Belgium; 4 Department of Medical Statistics, London School of Hygiene and Tropical Medicine, London, United Kingdom; 5 Centre for Clinical Infection and Diagnostics Research, Department of Infectious Diseases, King’s College London and Guy’s and St Thomas’ National Health Services Foundation Trust, London, United Kingdom; 6 Modelling and Economics Unit, National Infection Service, Public Health England, London, United Kingdom

**Keywords:** bacteremia, infection, excess length of stay, causal inference, confounding

## Abstract

**Background:**

Studies estimating excess length of stay (LOS) attributable to nosocomial infections have failed to address time-varying confounding, likely leading to overestimation of their impact. We present a methodology based on inverse probability–weighted survival curves to address this limitation.

**Methods:**

A case study focusing on intensive care unit–acquired bacteremia using data from 2 general intensive care units (ICUs) from 2 London teaching hospitals were used to illustrate the methodology. The area under the curve of a conventional Kaplan-Meier curve applied to the observed data was compared with that of an inverse probability–weighted Kaplan-Meier curve applied after treating bacteremia as censoring events. Weights were based on the daily probability of acquiring bacteremia. The difference between the observed average LOS and the average LOS that would be observed if all bacteremia cases could be prevented was multiplied by the number of admitted patients to obtain the total excess LOS.

**Results:**

The estimated total number of extra ICU days caused by 666 bacteremia cases was estimated at 2453 (95% confidence interval [CI], 1803–3103) days. The excess number of days was overestimated when ignoring time-varying confounding (2845 [95% CI, 2276–3415]) or when completely ignoring confounding (2838 [95% CI, 2101–3575]).

**Conclusions:**

ICU-acquired bacteremia was associated with a substantial excess LOS. Wider adoption of inverse probability–weighted survival curves or alternative techniques that address time-varying confounding could lead to better informed decision making around nosocomial infections and other time-dependent exposures.

## Teaser

This study illustrates how to estimate the excess length of stay due to nosocomial infections in the presence of time-varying confounding. The number of excess days was substantially overestimated when ignoring time-varying confounding, suggesting that future studies should adopt similar methodology.

Healthcare-associated infections (HCAIs) represent a public health problem and are associated with substantial morbidity, mortality, and costs [1–5]. A substantial proportion of HCAIs can be prevented by implementing infection prevention and control measures [4, 6–9].

While interventions may be effective, it is important to assess whether they are also cost-effective in the context of limited funds for healthcare. A key driver of the costs of HCAIs and—depending on the economic perspective, reimbursement system, and intervention—the cost-effectiveness of interventions targeting HCAI incidence is the excess length of stay (LOS) caused by these infections [6, 10]. To obtain useful estimates for policy making, studies need to take into account the timing of infection and the fact that patients who acquire an infection may not only differ at the day of admission, but also may deteriorate more before acquiring infection than patients who remain infection-free [11].

Common methodologies to account for the timing of infection are multistate models and Cox regression with infection implemented as a time-varying variable [12–14]. Applications of multistate models often do not adjust for baseline confounding and never correctly adjust for time-varying confounding (eg, severity of illness) [12, 15], which can limit their use. Furthermore, hazard ratios obtained from Cox regression models are often misinterpreted and do not readily translate into excess LOS in days [16, 17].

To address limitations of commonly applied methods, time-varying confounding can be adjusted for using marginal structural models fitted via inverse probability weighting (IPW) [11, 18]. In the presence of measured time-varying confounding, which is affected by past exposure, these and other g-methods have been shown to provide valid results where more conventional methodologies produce biased estimates [19–22].

When a time-varying confounder is also an intermediate variable on the causal pathway (eg, severity of illness is affected by the history of infection), adjusting for it would partly remove the effect of the exposure when using conventional regression techniques; it would moreover introduce collider-stratification bias, a form of selection bias that occurs when one controls for a common effect of exposure and outcome [11, 15, 23]. This has been empirically confirmed in simulation studies, which showed that a marginal structural model with IPW resulted in unbiased estimates of the effect of time-dependent harmful exposures on the hazard of hospital discharge [20]. In contrast, adjusting for time-varying confounding using a conventional Cox regression model with time-varying covariates, or freezing the time-varying confounder at their level before occurrence of the exposure, resulted in substantial bias [20].

Previous studies have not applied methods that correctly account for potential time-varying confounding when estimating excess LOS measured in days [15]. We recently used Cox proportional hazards regression to estimate what the observed risk of death in and discharge from the intensive care unit (ICU) would be if all patients in an ICU would remain bacteremia free compared to the actual situation where some patient do acquire bacteremia [11]. The corresponding survival curves could theoretically be combined to estimate the excess LOS. However, when the proportional hazards assumption holds conditional on a set of covariates, then it is almost guaranteed not to hold when one covariate is added or dropped. It is unlikely that one would have selected the precise set of covariates for which the proportional hazards assumption holds.

In view of this, we will instead make use of Kaplan-Meier (KM) curves along with IPW to predict what the LOS distribution would be if nosocomial infections could be prevented [24, 25]. Provided all confounders are measured and included in the model to create the inverse probability weights, one can assess excess LOS by comparing the observed LOS with that in the counterfactual situation, where all patients would remain infection-free. A major advantage of this method compared to several other approaches currently applied to estimate the excess LOS is that it simultaneously (*i*) does not suffer from bias due to ignoring the timing of infection; (*ii*) can address (measured, time-varying) confounding; (*iii*) does not require a proportional hazards assumption; (*iv*) has no built-in selection bias; and (*v*) produces excess LOS measured in days. We show using a case study how excess LOS due to time-dependent exposures such as ICU-acquired bacteremia can be estimated using KM estimators with IPW.

## METHODS

### Data

For our case study, we used data from all patients admitted to two 15-bed general ICUs at St Thomas’ Hospitals (London, United Kingdom) between January 2002 and March 2006 [11, 18]. Besides daily measurements on various invasive procedures and markers of severity of illness, these data contain information on admission, discharge, mortality, and administration of antimicrobial therapy [11]. Patients with an ICU LOS of < 3 days were excluded because patients could, by definition, only acquire bacteremia after being 2 days in the ICU. From the remaining cohort, we excluded patients with a blood culture positive for bacteria during the first 2 days in the ICU, to exclude community-acquired cases. After applying these exclusion criteria, a first blood culture positive for any bacteria obtained from the patient > 2 days after ICU admission was considered an ICU-acquired bacteremia. In line with other studies [1–3, 11–14, 18], only first infections were considered. Onset of infection was defined utilizing the time of the specimen taken as a proxy for time of infection.

The final model (Supplementary Material 1 for more details on all considered confounders and model selection) included age (restricted cubic spline with 2 degrees of freedom) and sex as baseline covariates and the Acute Physiology and Chronic Health Evaluation (APACHE) II score, receipt of any systemic antibiotic (yes/no), mechanical ventilation, and central lines as time-varying confounders [18]. For the APACHE score and antibiotic use, we used lagged values 2 days before to recognize that these variables measured within 24 hours before the onset of bacteremia are possible surrogate markers for an infection that was incubating and may therefore be affected by bacteremia [11, 18, 26]. Besides using these lagged values, we also adjusted for the cumulative previous time on mechanical ventilation and having received antibiotics (days on treatment). Furthermore, an interaction between the cumulative previous time on antibiotics and the cumulative previous time having received mechanical ventilation and an interaction between the presence of central lines on the first day of admission and the time-varying 1-day lagged presence of central lines were also included in the final model (see Supplementary Material 1, including sensitivity analyses using different lags).

### Analyses

We estimated the effect of bacteremia on LOS using several steps. We estimated the survival probabilities up to day 45 in the ICU for the observed data using conventional KM methodology. Subsequently we summed the survival probabilities for each day up to day *t* = 45 to obtain the average LOS up to day 45. This is the restricted mean survival time, which is equivalent to the area under the KM curve up to day 45 [27]. It can be interpreted as the average survival time within the interval of interest [27]. In line with others [2, 28], we used a cutoff of 45 days because the effect of bacteremia acquired later during the ICU stay may have a more limited impact and adjusting for potential time-varying confounding is more challenging for late infections, because there are simply insufficient numbers patients who acquire infection at the same time. Furthermore, only a very small proportion of patients stayed longer than 45 days in the ICU (3%), and the predicted probability of staying for at least 45 days in the ICU if bacteremia would be prevented was even lower (1.9%).

Subsequently, we treated bacteremia as a “censoring” event. Because patients who acquire an infection likely differ from patients who remain infection-free, a conventional KM curve based on these censored event times would be biased. We adjusted for this bias using IPW [29]. The weights were constructed using pooled logistic regression models for the probability of censoring on each day, in this case acquiring bacteremia [11]. Confounding was removed by reweighting patients in the risk set (those who were still present in the hospital and not artificially censored because of bacteremia) on each day [11, 19].

KM methodology with IPW was subsequently applied to the data. Subsequently, the survival probabilities for each day up to day *t* = 45 were summed to obtain the average LOS in the absence of bacteremia.

The difference between the average observed LOS and the average LOS that would be observed if all bacteremias could be prevented was multiplied by the number of admitted patients, to obtain the total excess LOS attributable to ICU-acquired bacteremias. We estimated 95% confidence intervals (CIs) as described in Supplementary Material 2.

The effect per infection can be obtained by dividing the difference between the average observed LOS and the counterfactual average LOS in the absence of infection by the probability of acquiring an infection during the ICU stay. All analyses were performed using R version 3.5.1 software (R code in Supplementary Material 3).

## RESULTS

**Figure 1.**
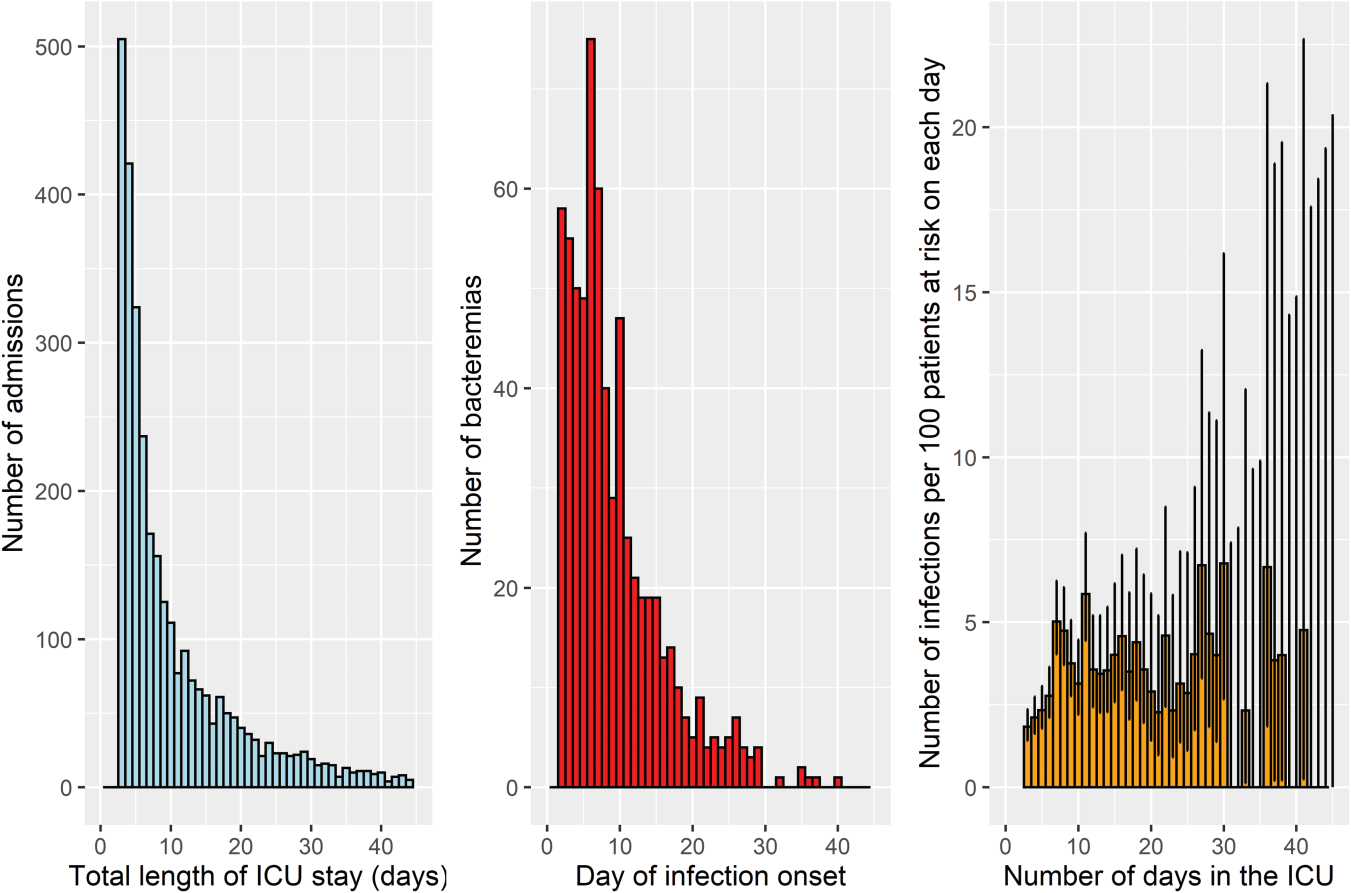
Distributions of total length of intensive care unit stay (left), day of bacteremia onset (middle), and the probability of acquiring bacteremia on each day (right). The error bars in the right panel represent 95% confidence intervals based on Wilson score intervals. Abbreviation: ICU, intensive care unit.

In total 2914 patients were admitted to the ICU and stayed > 2 days in the ICU, contributing to 3157 ICU admissions. Table 1 shows the characteristics of included admissions. Most patients had a relatively short total LOS (median, 7 days [25th–75th percentile, 4–15]), resulting in a highly skewed distribution (Figure 1, left panel). As mentioned earlier, patients were only included if they stayed > 2 days in the ICU; however, the first 2 days were added to the average LOS. The observed average LOS in the ICU was 11.44 (95% CI, 11.07–11.81) days when restricting the follow-up to the first 45 days after admission. There were 666 blood cultures positive for bacteria, with coagulase-negative staphylococci (44%), mixed bacteria (17%), *Staphylococcus aureus* (8%), *Pseudomonas aeruginosa* (6%), and *Escherichia coli* (4%) being the most commonly isolated bacteria. The majority (82%) of the 666 ICU-acquired bacteremias occurred within the first 2 weeks after admission (Figure 1, middle panel). However, among those still at risk of acquiring a bacteremia, the daily probability of acquiring it initially increased over time (Figure 1, right panel). The width of the CIs around these probabilities increased over time due to the decreasing number of patients still at risk, precluding conclusions about the risk of bacteremia for long-stay patients.

**Table 1.** Characteristics of Patients for Intensive Care Unit Admissions of > 2 Days During the First 3–45 Days After Admission

Characteristic	Admissions (N = 3157)^a^
Male sex, No. (%)	1915 (61)
Age, y, median (25th–75th percentile)	64 (50–74)
APACHE score at admission, median (25th–75th percentile)	18 (14–22)
Central lines at admission, No. (%)	2182 (69)
No. of antibiotics at admission, median (25th–75th percentile)	0 (0–2)
Mechanical ventilation at admission, No. (%)	2376 (75)
Died in ICU, No. (%)	626 (20)

Abbreviations: APACHE, Acute Physiology and Chronic Health Evaluation; ICU, intensive care unit.

^a^Given that patients with an ICU stay < 3 days were excluded, the percentage of patients receiving invasive procedures is likely larger than for the average of all patients admitted to the ICU.

### Inverse Probability–Weighted Survival Curves

Details of the pooled logistic models used to create the inverse probability weights are provided in the Supplementary Material. The weights had a median and mean of 1.00 and 1.01, respectively, and an interquartile range and standard deviation of 0.06 and 0.13 (range, 0.44– 2.09), respectively. The values raise no concern that our results may be affected by extreme weights [29].

If all 666 ICU-acquired bacteremias would be prevented, the inverse probability–weighted KM curve predicted that patients would stay on average 10.66 (95% CI, 10.27–11.06) days when restricting the follow-up to the first 45 days after admission. The estimated total number of extra days in the ICU due to bacteremia were estimated at 2453 (95% CI, 1803–3103) days. The estimated number of excess days per infection were 3.7 days $\left(11.439-10.662\right)*\;{\left(\frac{666}{3157}\right)}^{-1}$.

### Influence of Confounding

When ignoring confounding, using a weight of 1 for each observation, the number of excess days was estimated to be higher during the study period at 2838 (95% CI, 2101–3575) days. Similarly, the number of excess days was substantially overestimated when taking only confounders measured at admission into account, with an estimated number of 2845 (95% CI, 2276–3415) days attributable to infection.

## DISCUSSION

In this article, we illustrated how to estimate the excess LOS associated with time-dependent exposures, such as ICU-acquired bacteremia, in the presence of time-varying confounding. The number of excess days due to ICU-acquired bacteremia was substantially overestimated when ignoring time-varying confounding, suggesting that future studies should adopt the methodology presented here or alternative techniques [22, 23] that correctly address time-varying confounding without inducing collider-stratification bias or removing the indirect effects of early infection on the LOS that are mediated through the considered confounders.

Inverse probability–weighted KM methodology has appealing features compared to other techniques used in the literature. This includes the ability to simultaneously address the time-dependent nature of healthcare-acquired infections and time-invariant and time-varying confounding, as well as producing excess LOS estimates measured in days. Moreover, in contrast to many other approaches, no (semi-)parametric assumptions are needed on the distribution of LOS. The time-dependent nature of ICU-acquired bacteremia was taken into account using IPW based on the daily probability of acquiring bacteremia among those still at risk. Other studies have addressed the time-dependent nature of nosocomial infections using multistate models, without accounting for time-varying confounding [1–3]. Given that one would expect that patients who acquire a bacteremia may be more severely ill and have deteriorated more than patients who remain infection-free, these previous studies have likely resulted in confounded estimates, thereby potentially overestimating the effect on excess LOS. While there are some recent studies that used g-methods such as IPW to address time-varying confounding to assess the effect of infections on ICU mortality and ICU discharge [11, 18, 26, 30, 31], to our knowledge this is the first study that uses similar methodology to estimate the effect of time-varying exposures on excess LOS.

While we focused on ICU-acquired bacteremia, the methodology proposed here can be applied to address any research question on the attributable effect of a (dichotomous) time-varying exposure on an uncensored event time. Whenever disease severity influences the decision to initiate drug therapy, time-varying confounding is likely [20]. If such confounding is influenced by prior exposure, thus also being an intermediate on the causal pathway, conventional regression approaches will provide biased estimates by removing part of the effect of the exposure and introducing selection bias. If there are competing events or other forms of censoring, our methodology can still be used if censoring is more broadly defined by treating both the exposure of interest and competing risks as censoring events, provided that there is no unmeasured confounding.

Our results add to the growing literature showing that ignoring confounding or only adjusting for confounders measured at baseline could result in substantial bias [11, 19, 20, 26, 30, 31]. In any case, evaluating whether (time-varying) confounding might play a role is preferable over directly applying multistate or other models without such adjustment, as such approaches implicitly assume that time-varying confounding can be ignored.

There are several limitations to our study. Regarding our case study, the estimates of the total excess number of ICU days due to ICU-acquired bacteremia rely on accurate measurements of all relevant confounders. We did not have information on some potential time-varying confounders such as surgeries or abdominal perforation and baseline confounders such as main reason for admission, which may both increase the risk of infection as well as the risk of death independent of infection. Therefore, we may have overestimated the effect of ICU-acquired bacteremia and underestimated the impact of not accounting for all time-varying confounders, thereby further strengthening the case for collection and correct adjustment for time-varying confounders in future studies. When creating the pooled logistic regression models that are used for estimation of the weights, it is important to consider potential relevant interactions and cumulative measures of daily measurements as these typically capture the evolution of severity of illness better than simple daily measurements alone.

The causal inference literature highlights that exchangeability (implying no unmeasured confounding, discussed above), positivity (the probability of acquiring bacteremia or remaining bacteremia-free being positive for every individual), and consistency (informally the need for a well-defined [hypothetical] intervention) are necessary conditions for causal inference [30]. The mean of our estimated weights was 1.00 and there were no extreme values, thereby raising no concern about violation of the positivity assumption [29]. The need for a well-defined intervention (consistency assumption) has been highly debated in the literature [32–34], with common misconceptions being that the hypothetical intervention needs to be currently feasible, or that the net effect of the exposure of interest cannot be estimated if there is not a single well-defined (hypothetical) intervention that could be used to alter the exposure [33, 34]. Specifically for our study, the results can be viewed as providing an estimate of the harmful net effect of ICU-acquired bacteremia. This effect likely differs from the effect of an intervention to prevent ICU-acquired bacteremia because it is unlikely that such intervention would just prevent the development of bacteremia without having any other effects [33].

Onset of infection was defined using the time the first positive specimen was taken; however, it is uncertain whether onset may have occurred earlier. We used lagged values measured from the previous day to predict infection (and 2 days for the APACHE score and antibiotic treatment) to prevent adjustment for postinfection measurements.

Some positive blood cultures may have actually been contaminated blood samples (eg, some of those positive for coagulase-negative staphylococci), thereby potentially leading to an underestimation of the effect of true bacteremia. Of note, this dataset was from before widespread implementation of infection control bundles for vascular catheter care, when coagulase-negative staphylococci infections were more frequent and significant than now. The proportion of bacterial infections that are resistant to commonly used antibiotics has increased since the end of the study period and antibiotic resistance has been associated with increases in LOS [2, 3]. While these studies estimating the effect of resistance on LOS did not adjust for (time-varying) confounding, the current effect of ICU-acquired bacteremia may be larger than the estimate from this study.

We focused on bacteremias in general, which is a combination of all different types of bacteria with different antibiotic resistance profiles causing bacteremia. The effect of specific bacteria and associated resistances could be estimated in larger multicenter studies using similar methodology.

Finally, we could obtain the average effect per infection using the IPW KM methodology. However, this approach does not provide the excess LOS due to acquisition of an infection on a given day after admission, which is potentially easier to interpret given the likely varying effects of acquisition of infections early after admission or much later during the ICU stay.

In summary, ICU-acquired bacteremia is associated with a substantial number of excess days in the ICU, even after adjusting for time-varying confounding. Inverse probability–weighted KM curves form a relatively simple method to estimate the causal effect of time-varying exposures on excess length of hospital stay. Wider adoption of this, or other techniques that correctly address time-varying confounding, could lead to better-informed decision making.

## Supplementary Data

Supplementary materials are available at *Clinical Infectious Diseases* online. Consisting of data provided by the authors to benefit the reader, the posted materials are not copyedited and are the sole responsibility of the authors, so questions or comments should be addressed to the corresponding author.

ciaa136_suppl_Supplementary_Material_1

ciaa136_suppl_Supplementary_Material_2

ciaa136_suppl_Supplementary_Material_3

## Notes

***Acknowledgments.*** The Improving the uptake and Sustainability of Effective interventions to promote Prudent antibiotic Use in Primary care (STEP-UP) team consists of Philip E. Anyanwu, Aleksandra Borek, Nicole Bright, James Buchanan, Christopher Butler, Anne Campbell, Ceire Costelloe, Benedict Hayhoe, Alison Holmes, Susan Hopkins, Azeem Majeed, Monsey McLeod, Michael Moore, Liz Morrell, Koen B. Pouwels, Julie V. Robotham, Laurence S. J. Roope, Sarah Tonkin-Crine, Ann Sarah Walker, Sarah Wordsworth, and Anna Zalevski.

***Financial support.*** This work was supported by the Economic and Social Research Council through the Antimicrobial Resistance Cross Council Initiative supported by the 7 research councils in partnership with other funders (grant number ES/P008232/1). K. B. P. is supported by the National Institute for Health Research (NIHR) Health Protection Research Unit in Healthcare Associated Infections and Antimicrobial Resistance at the University of Oxford in partnership with Public Health England (grant number HPRU-2012–10041). This research was supported by the NIHR Biomedical Research Centre based at Guy’s and St Thomas’ National Health Service (NHS) Foundation Trust and King’s College London Programme of Infection and Immunity (RJ112/N027) awarded to J. E., and the NIHR Collaboration for Leadership in Applied Health Research and Care South London at King’s College Hospital NHS Foundation Trust, awarded to J. E. and R. B.

***Potential conflicts of interest.*** The authors: No reported conflicts of interest. All authors have submitted the ICMJE Form for Disclosure of Potential Conflicts of Interest.
