# Supplementary material for: Estimating the Effect of Healthcare-Associated Infections on Excess Length of Hospital Stay Using Inverse Probability–Weighted Survival Curves
Source: Clin Infect Dis. 2020 Feb 12;71(9):e415–20. doi: 10.1093/cid/ciaa136 (PMC7713691; doi:10.1093/cid/ciaa136)
Supplement: ciaa136_suppl_Supplementary_Material_1 [file ciaa136_suppl_supplementary_material_1.docx]

**Supplementary Material** **1: Model to create inverse probability weights**

**Estimating the effect of healthcare-associated infections on excess length of hospital stay using inverse-probability weighted survival curves**

**Koen B. Pouwels, Stijn Vansteelandt, Rahul Batra, Jonathan Edgeworth, Sarah Wordsworth,**

**Julie Robotham, on behalf of the STEP-UP team.**

*Model to create inverse probability weights*

Daily probabilities of acquiring bacteraemia in the ICU were estimated using a pooled logistic regression model (logistic regression with each day within a patient treated as a separate observation). For the current analyses, we included age (using a restricted cubic spline with 3 degrees of freedom) and sex as baseline covariates and the Acute Physiology and Chronic Health Evaluation (APACHE) II score, receipt of systemic antimicrobials, mechanical ventilation and central lines as potential time-varying confounders.

Because time-varying variables measured on a given day may have been influenced by infection acquired on that day, lagged values from the day before were included in the models. For the APACHE II score and antibiotic use, we adjusted for lagged values two days before to acknowledge that the APACHE II score and antibiotic use within 24 h before the onset of bacteremia are potentially surrogate markers for an infection that was incubating and hence may be affected by bacteremia on that day.

Furthermore, to take into account better trajectories of severity of illness over time, the cumulative previous time on mechanical ventilation, having central lines and having received antibiotics were considered as potential confounders. In addition, we allowed for interactions between measurements at admission and during follow-up for all time-varying variables, e.g. whether the patient received a central line on the first day of admission interacting with the time-varying 1-day lagged presence of central line(s). Two-way interactions between cumulative time-varying variables (cumulative days of antibiotics, mechanical ventilation, and central lines) were also considered, e.g. cumulative previous time on antibiotics interacting with the cumulative previous time having received mechanical ventilation. Above interactions were considered for inclusion based on potential clinical relevance and their potential to capture potential changes and patterns in severity of illness.

All variables described above were considered potential confounders or markers of confounders such as severity of illness. Inclusion in the final model was based on goodness of fit of the model (Akaike Information Criterion). Main effects were selected based on backward selection after which potential pre-specified interactions (see above) were added to the model (including adding the main effects back in again if they were removed before if necessary). Variables that were included in the final model were: sex, age (modelled using a restricted cubic spline with 2 degrees of freedom), APACHE II score at admission, presence of central lines at admission, mechanical ventilation at admission, number of systemic antibiotics at admission, 2-day lagged value of APACHE score, 1-day lagged presence of central lines, 1-day lagged value of presence of mechanical ventilation, 2-day lagged value of systemic antibiotics, cumulative previous time on antibiotics (lagged by 2 days), cumulative previous time on mechanical ventilation (lagged by 1 day and modelled using a restricted cubic spline with 5 degrees of freedom). Furthermore, the final model included an interaction between the cumulative previous time on antibiotics (lagged by 2 days) and the cumulative previous time having received mechanical ventilation (lagged by 1 day), and an interaction between the presence of central lines on the first day of admission and the time-varying 1-day lagged presence of central lines.

It should be noted that the coefficients from this model should not be given a causal interpretation, as the model is merely used to create a statistical population in which bacteraemia on each day is independent of measured daily indicators of disease severity. The obtained probabilities were used to generate daily patient-specific inverse probability weights.

**Sensitivity analysis:**

Sensitivity analyses evaluating whether the results were sensitive to the choice of the lags for potential confounders were performed with i) lagged values of 1 day for all potential confounders or ii) with lagged values of 3 days for the APACHE II score and (cumulative) antibiotic use. These sensitivity analyses gave identical results when rounded to one decimal.
