# Supplementary material for: Estimating the Effect of Healthcare-Associated Infections on Excess Length of Hospital Stay Using Inverse Probability–Weighted Survival Curves
Source: Clin Infect Dis. 2020 Feb 12;71(9):e415–20. doi: 10.1093/cid/ciaa136 (PMC7713691; doi:10.1093/cid/ciaa136)
Supplement: ciaa136_suppl_Supplementary_Material_2 [file ciaa136_suppl_supplementary_material_2.pdf]

Supplementary file 2: Confidence intervals for excess days

Estimating the effect of healthcare-associated infections on excess length of hospital stay using inverse-probability weighted survival curves

Koen B. Pouwels, Stijn Vansteelandt, Rahul Batra, Jonathan Edgeworth, Sarah Wordsworth, Julie Robotham, on behalf of the STEP-UP team.

1 Standard error of average length of stay

Let d_t be the number of events on day t and n_t be the number of people at risk on day t; throughout, we let t take values only over the time points at which measurements are obtained, e.g. t runs over the different days in ICU. Then the hazard on day t can be estimated as $\hat{\lambda}_t = d_t/n_t$. The log survival probability on day t is then estimated as

$$\log \hat{S}_t = \sum_{j=1}^{t} \log\left(1 - \hat{\lambda}_j\right).$$

The average length of stay over the time window from day 1 to day T is then estimated as

$$\hat{L} = \sum_{t=1}^{T} \hat{S}_t.$$

First note that

$$\begin{aligned}
\text{Var}\left(\log \hat{S}_t\right) &= \sum_{j=1}^{t} \text{Var}\left\{\log\left(1 - \hat{\lambda}_j\right)\right\} \\
&\approx \sum_{j=1}^{t} \text{Var}\left(\hat{\lambda}_j\right) / \left(1 - \hat{\lambda}_j\right)^2 \\
&= \sum_{j=1}^{t} \hat{\lambda}_j\left(1 - \hat{\lambda}_j\right) / \left(1 - \hat{\lambda}_j\right)^2 \\
&= \sum_{j=1}^{t} \frac{d_j}{n_j(n_j - d_j)}.
\end{aligned}$$

Further, for $s < t$,

$$\begin{aligned}\text{Cov}\left(\log \hat{S}_s, \log \hat{S}_t\right) &= \text{Var}\left(\log \hat{S}_s\right) \\ &= \sum_{j=1}^{s} \frac{d_j}{n_j(n_j - d_j)}.\end{aligned}$$

Let $\hat{S}$ be the column vector $\left(\hat{S}_1, ..., \hat{S}_T\right)'$, where a' refers to the transpose of a. Let Γ be the diagonal matrix with diagonal $\hat{S}$, and Σ be a symmetric matrix with diagonal elements $\text{Var}\left(\log \hat{S}_t\right)$ and off-diagonal element $\text{Cov}\left(\log \hat{S}_s, \log \hat{S}_t\right)$ in row s and column $t > s$. Then by the delta method, the variance-covariance matrix of $\hat{S}$ can be estimated as

$$\Gamma\Sigma\Gamma.$$

Further, let P be a column vector of length T with all elements equalling 1. Then the variance of the average length of stay $\hat{L}$ can be estimated as

$$P'\Gamma\Sigma\Gamma P.$$

The standard error is its square root.

2 Standard error of the counterfactual average length of stay

Throughout, I will assume that the inverse probability weights are known. Let d_t^w be the weighted number of events on day t and n_t^w be the weighted number of people at risk on day t. Then the counterfactual hazard on day t can be estimated as $\hat{\lambda}_t^w = d_t^w / n_t^w$. The counterfactual log survival probability on day t is then estimated as

$$\log \hat{S}_t^w = \sum_{j=1}^{t} \log\left(1 - \hat{\lambda}_j^w\right).$$

The counterfactual average length of stay over the time window from day 1 to day T is then estimated as

$$\hat{L}^w = \sum_{t=1}^{T} \hat{S}_t^w.$$

Since we continue to have independence of the number of events across risk sets when the weights are known, the previous formulas continue to apply. In particular, since $\hat{\lambda}_j^w$ is a weighted average with known weights, we have that

$$\text{Var}\left(\hat{\lambda}_t^w\right) = \lambda_t(1 - \lambda_t) N_t^w / (n_t^w)^2,$$

where N_t^w denotes the sum of the squared weights in the risk set at time t. Note that the above expression uses λ_t and NOT λ_t^w! We thus find that

$$\begin{aligned}\text{Var}\left(\log \hat{S}_t^w\right) &= \sum_{j=1}^{t} \frac{\hat{\lambda}_j\left(1-\hat{\lambda}_j\right)N_j^w}{(n_j^w)^2\left(1-\hat{\lambda}_j^w\right)^2} \\ &= \sum_{j=1}^{t} \frac{d_j(n_j-d_j)N_j^w}{n_j^2(n_j^w-d_j^w)^2},\end{aligned}$$

and for $s < t$,

$$\text{Cov}\left(\log \hat{S}_s^w, \log \hat{S}_t^w\right) = \text{Var}\left(\log \hat{S}_s^w\right).$$

Let $\hat{S}^w$ be the column vector $\left(\hat{S}_1^w, ..., \hat{S}_T^w\right)'$. Let Γ^w be the diagonal matrix with diagonal $\hat{S}^w$, and Σ^w be a symmetric matrix with diagonal elements $\text{Var}\left(\log \hat{S}_t^w\right)$ and off-diagonal element $\text{Cov}\left(\log \hat{S}_s^w, \log \hat{S}_t^w\right)$ in row s and column $t > s$. Then by the delta method, the variance-covariance matrix of $\hat{S}^w$ can be estimated as

$$\Gamma^w \Sigma^w \Gamma^w.$$

Further, let P be a column vector of length T with all elements equalling 1. Then the variance of the average length of stay $\hat{L}^w$ can be estimated as

$$P' \Gamma^w \Sigma^w \Gamma^w P.$$

The standard error is its square root.

The previous calculation assumes that the weights w_{it} are deterministic. Using the delta method, we can acknowledge their stochastic nature. In particular, we can linearize

$$\hat{\lambda}_t^w - \lambda_t^w \approx \frac{1}{n_t^w} \sum_{i=1}^{n_t} w_{it}(\delta_{it} - \hat{\lambda}_t^w),$$

where we can view n_t^w and $\hat{\lambda}_t^w$ as constants, and where δ_{it} equals 1 for subjects who experience an event at time t and 0 otherwise, and w_{it} is the weight for individual i on day t. We then find that

$$\text{Var}\left(\hat{\lambda}_t^w\right) = \frac{1}{(n_t^w)^2} \sum_{i=1}^{n_t} \left\{w_{it}(\delta_{it} - \hat{\lambda}_t^w)\right\}^2.$$

We thus find that

$$\text{Var}\left(\log \hat{S}_t^w\right) = \sum_{j=1}^{t} \frac{1}{(n_j^w)^2\left(1-\hat{\lambda}_j^w\right)^2} \sum_{i=1}^{n_j} \left\{w_{ij}(\delta_{ij} - \hat{\lambda}_j^w)\right\}^2$$

and for $s < t$,

$$\text{Cov}\left(\log \hat{S}_s^w, \log \hat{S}_t^w\right) = \text{Var}\left(\log \hat{S}_s^w\right).$$

With these modifications, the variance of the average length of stay $\hat{L}^w$ can be calculated as before.

3 Standard error of the difference between observed and counterfactual average length of stay

To infer the standard error of the difference between observed and counterfactual average length of stay, we also need to know the covariance between the estimators $\log \hat{S}_s$ and $\log \hat{S}_t^w$ for $s \neq t$. For $s \leq t$, we have that

$$\begin{aligned}
\text{Cov}\left(\log \hat{S}_s, \log \hat{S}_t^w\right) &= \text{Cov}\left(\log \hat{S}_s, \log \hat{S}_s^w\right) \\
&= \sum_{j=1}^{s} \text{Cov}\left\{\log\left(1-\hat{\lambda}_j\right), \log\left(1-\hat{\lambda}_j^w\right)\right\} \\
&\approx \sum_{j=1}^{s} \frac{\text{Cov}\left(\hat{\lambda}_j, \hat{\lambda}_j^w\right)}{\left(1-\hat{\lambda}_j\right)\left(1-\hat{\lambda}_j^w\right)}.
\end{aligned}$$

Then

$$\begin{aligned}
\text{Cov}\left(\hat{\lambda}_j, \hat{\lambda}_j^w\right) &= \text{Cov}\left(n_j^{-1}\sum_{i=1}^{n_j}\delta_{ij}, n_j^{w,-1}\sum_{i=1}^{n_j} w_{ij}\delta_{ij}\right) \\
&= \frac{1}{n_j n_j^w}\sum_{i=1}^{n_j} \text{Cov}\left(\delta_{ij}, w_{ij}\delta_{ij}\right) \\
&= \frac{1}{n_j n_j^w}\sum_{i=1}^{n_j} w_{ij}\text{Var}(\delta_{ij}) \\
&= \frac{1}{n_j}\hat{\lambda}_j(1-\hat{\lambda}_j)
\end{aligned}$$

from which

$$
\begin{aligned}
\mathrm{Cov}\left(\log \hat{S}_s, \log \hat{S}_t^w\right) &= \mathrm{Cov}\left(\log \hat{S}_s, \log \hat{S}_s^w\right) \\
&\approx \sum_{j=1}^{s} \frac{\hat{\lambda}_j(1-\hat{\lambda}_j)}{n_j\left(1-\hat{\lambda}_j\right)\left(1-\hat{\lambda}_j^w\right)} \\
&= \sum_{j=1}^{s} \frac{\hat{\lambda}_j}{n_j\left(1-\hat{\lambda}_j^w\right)} \\
&= \sum_{j=1}^{s} \frac{d_j n_j^w}{n_j^2\left(n_j^w - d_j^w\right)}.
\end{aligned}
$$

For $s > t$, we have that

$$
\begin{aligned}
\mathrm{Cov}\left(\log \hat{S}_s, \log \hat{S}_t^w\right) &= \mathrm{Cov}\left(\log \hat{S}_t, \log \hat{S}_t^w\right) \\
&\approx \sum_{j=1}^{t} \frac{d_j n_j^w}{n_j^2\left(n_j^w - d_j^w\right)}.
\end{aligned}
$$

We are now ready to calculate a standard error on $\hat{L} - \hat{L}^w$. Let $\hat{S}^f$ be the column vector $\left(\hat{S}', \hat{S}^{w\prime}\right)'$. Let Γ^f be the diagonal matrix with diagonal $\hat{S}^f$, and Σ^f be a symmetric matrix with upper left block Σ, bottom right block Σ^f, and in the remain blocks the elements $\mathrm{Cov}\left(\log \hat{S}_s, \log \hat{S}_t^w\right)$. In particular, in row $s = 1, ..., T$ and column $T + s + t$ for $t = 0, ..., T - s$ we place the element $\mathrm{Cov}\left(\log \hat{S}_s, \log \hat{S}_{s+t}^w\right)$. Then by the delta method, the variance-covariance matrix of $\hat{S}^f$ can be estimated as

$$\Gamma^f \Sigma^f \Gamma^f.$$

Further, let P^f be a column vector of length $2T$ with the first T elements equalling 1 and the last T elements equalling -1 (corresponding to taking the difference $\hat{L} - \hat{L}^w$). Then the variance of $\hat{L} - \hat{L}^w$ can be estimated as

$$P^{f\prime} \Gamma^f \Sigma^f \Gamma^f P^f.$$

The standard error is its square root.

The previous calculation assumes that the weights w_{it} are deterministic. Linearizing $\hat{\lambda}_t^w$ as in the previous section, we find that

$$
\begin{aligned}
\mathrm{Cov}\left(\hat{\lambda}_j, \hat{\lambda}_j^w\right) &= \mathrm{Cov}\left(n_j^{-1}\sum_{i=1}^{n_j} \delta_{ij}, \frac{1}{n_j^w}\sum_{i=1}^{n_j} w_{ij}(\delta_{ij} - \hat{\lambda}_j^w)\right) \\
&= \frac{1}{n_j n_j^w}\sum_{i=1}^{n_j} \delta_{ij} w_{ij}(1 - \hat{\lambda}_j^w).
\end{aligned}
$$

For $s \leq t$, we then have that

$$\begin{aligned} \mathrm{Cov}\left(\log \hat{S}_s, \log \hat{S}^w_t\right) &= \mathrm{Cov}\left(\log \hat{S}_s, \log \hat{S}^w_s\right) \\ &\approx \sum_{j=1}^{s} \frac{1}{n_j n^w_j \left(1-\hat{\lambda}_j\right)\left(1-\hat{\lambda}^w_j\right)} \sum_{i=1}^{n_j} \delta_{ij} w_{ij} (1-\hat{\lambda}^w_j). \end{aligned}$$

For $s > t$, we have that

$$\begin{aligned} \mathrm{Cov}\left(\log \hat{S}_s, \log \hat{S}^w_t\right) &= \mathrm{Cov}\left(\log \hat{S}_t, \log \hat{S}^w_t\right) \\ &\approx \sum_{j=1}^{t} \frac{1}{n_j n^w_j \left(1-\hat{\lambda}_j\right)\left(1-\hat{\lambda}^w_j\right)} \sum_{i=1}^{n_j} \delta_{ij} w_{ij} (1-\hat{\lambda}^w_j). \end{aligned}$$

With these modifications, the variance of the average length of stay $\hat{L}^w$ can be calculated as before.
